# Hide and Seek: Nanomaterial Interactions With the Immune System

**DOI:** 10.3389/fimmu.2019.00133

**Published:** 2019-02-01

**Authors:** Bengt Fadeel

**Affiliations:** Nanosafety and Nanomedicine Laboratory, Institute of Environmental Medicine, Karolinska Institutet, Stockholm, Sweden

**Keywords:** nanomaterials, immune system, stealth, biomimetic, bio-corona, targeting

## Abstract

Engineered nanomaterials hold promise for a wide range of applications in medicine. However, safe use of nanomaterials requires that interactions with biological systems, not least with the immune system, are understood. Do nanomaterials elicit novel or unexpected effects, or is it possible to predict immune responses to nanomaterials based on how the immune system handles pathogens? How does the bio-corona of adsorbed biomolecules influence subsequent immune interactions of nanomaterials? How does the grafting of polymers such as poly(ethylene glycol) onto nanomaterial surfaces impact on these interactions? Can ancient immune evasion or “stealth” strategies of pathogens inform the design of nanomaterials for biomedical applications? Can nanoparticles co-opt immune cells to target diseased tissues? The answers to these questions may prove useful for the development of nanomedicines.

## Introduction

Engineered nanomaterials offer exciting opportunities for diagnosis and therapy of human disease. However, it is mandatory to address whether undesirable interactions occur between nanoparticles (NPs) and the nanoscale machineries of biological systems (1, 2). One may ask whether NPs elicit any novel or unexpected effects, or whether immune responses toward such materials are conserved and, therefore, predictable, on the basis of our knowledge of immune responses to viruses and other pathogens? This has considerable implications for nanomedicine. The immune system has evolved to protect us from foreign intrusion (pathogens, particles) and from internal “danger” (3), but immune responses can also be exploited for therapeutic gain (4). It may or may not be desirable for nanomaterials to engage with the immune system depending on the intended application (5). Here, a brief discussion of immune recognition and immune evasion of NPs is provided, along with the role of the bio-corona in modulating these interactions. The potential for targeting of nanoparticles in the context of nanomedicine is also discussed. The main thesis is that valuable lessons can be learned from the study of immune responses to viruses and other pathogens (6).

## Immune Sensing

Engineered nanomaterials are readily internalized by phagocytes of the innate immune system (7). The question is whether this uptake is mediated through specific receptors: are pattern recognition receptors (PRRs) that have evolved in order to sense invading pathogens moonlighting as receptors for engineered nanomaterials, or is the uptake non-specific? Furthermore, are nanomaterials immunologically inert or do they elicit (specific) immune responses? In other words, are nanomaterials immunogenic? The term *immunogenicity* refers to the ability of a substance to induce a cellular and/or humoral (antibody-mediated) immune response, whereas *antigenicity* is the ability to bind specifically to T cell receptors or antibodies (B cell receptors) induced as a result of an immune response (8). Hence, while all immunogenic substances are antigenic, not all antigenic substances are immunogenic. Two decades ago, the discovery of antibodies specific for fullerenes was reported (9). Ten years earlier, antibodies to cholesterol crystals were obtained, though cholesterol was widely regarded as a poorly immunogenic substance at the time (10). These findings suggested that the immune system recognizes repetitive patterns reminiscent of those present on (nano-sized) viruses, and testified to the remarkable capacity of the immune system to generate antibodies against virtually any chemical species, natural or synthetic (11). Interestingly, Erlanger et al. (12) could show that antibodies specific for fullerenes also bind single-walled carbon nanotubes (SWCNTs). In another study, an antibody fragment with high affinity and selectivity for gold surfaces was identified (13). However, as pointed out recently (8), NP conjugation to a protein carrier is usually required for successful antibody induction, and NPs tend to behave as haptens (i.e., small molecules that elicit an immune response only when attached to a carrier such as a protein). Nevertheless, as nanomaterials rapidly associate with proteins when they enter into the body (14), close attention to the potential immunogenicity of nanomaterials is necessary. Furthermore, antibodies against the surface coating of nanomaterials, including poly(ethylene glycol) (PEG), are also important to consider (15). To add to the complexity, metal/metal oxide NPs may undergo dissolution with the release of metal ions, and though this is recognized as one potential mechanism of nanotoxicity, there are few studies on the immunogenic role of the released ions. For comparison, chronic beryllium disease, a fibrotic lung disorder caused by exposure to beryllium (Be), is characterized by the accumulation of Be-responsive CD4^+^ T cells in the lung (16). Notably, these T cells are not directed to Be itself; instead, Be^2+^ ions induce a conformational change in certain HLA-DP2-peptide complexes leading to their recognition as neoantigens (17). These findings blur the distinction between hypersensitivity (to metals) and autoimmunity. Whether or not other metal ions released from metallic (nano)particles may exert similar effects deserves to be studied.

**Figure 1:**
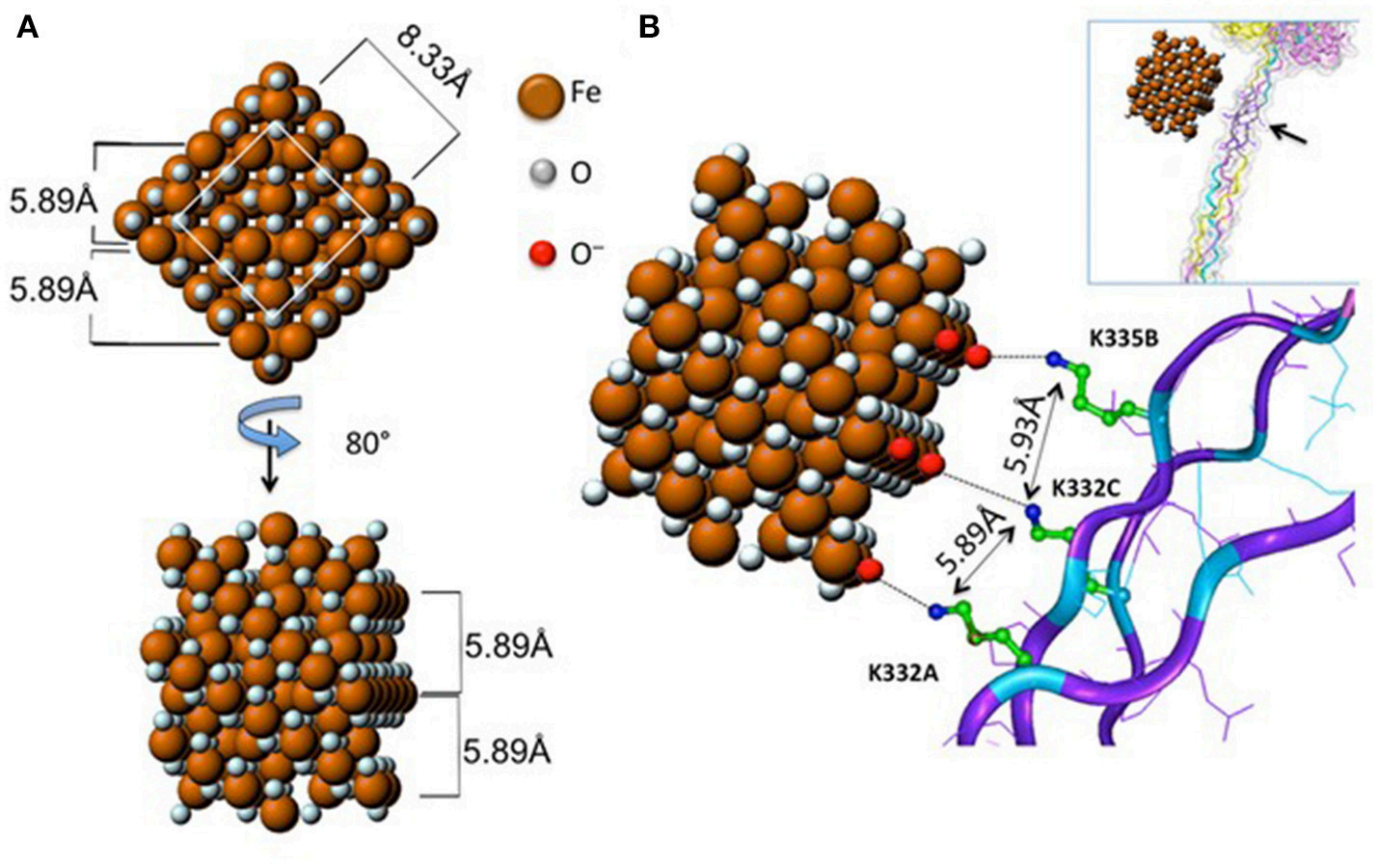
Receptor recognition of nanoparticles. Proposed model of the interaction between the scavenger receptor, SR-AI, a so-called pattern recognition receptor, and crystalline magnetite. **(A)** 2D frontal and rotated projections of the magnetite unit cell. **(B)** 3D view of the crystal unit approaching the charged collagen-like domain of SR-A1. Reprinted with permission from Chao et al. (28). Copyright (2013) American Chemical Society.

Do nanomaterials exploit specific receptors to gain entry into macrophages or other immune cells? Scavenger receptors were originally identified based on their ability to recognize and to remove modified lipoproteins, but this heterogenous family of receptors is now known to recognize a diverse range of ligands (18). Soluble extracellular domains of scavenger receptors were found to bind crocilodite asbestos (19). Furthermore, the scavenger receptor, MARCO (macrophage receptor with collagenous structure) has been shown to mediate the ingestion of micron-sized environmental particles by alveolar macrophages (20). Moreover, polystyrene NPs and silica NPs also bind to MARCO (21, 22). However, the overexpression of scavenger receptors in non-phagocytic cell lines may not reflect their actual role in primary macrophages. We recently demonstrated that the class A scavenger receptor (SR-A1) as well as the mannose receptor CD206, two well-known PRRs, are deployed by primary human macrophages for uptake of mesoporous silica particles (23). In another recent study, Tsugita et al. (24) identified the class B scavenger receptor, SR-B1 as a receptor for both amorphous and crystalline silica, but not TiO_2_ NPs, or monosodium urate crystals, although each of these ligands exhibited negative surface potentials. The latter finding suggested that SR-B1 recognizes not only the electrostatic potential of the silica surface, but also molecular determinants within silica, through interactions with specific residues. The authors also showed that SR-B1-mediated recognition of silica is associated with canonical inflammasome activation (24). Furthermore, we have recently shown that endotoxin-free SWCNTs can signal *via* Toll-like receptors (TLRs), leading to a TLR/MyD88/NF-κB-dependent macrophage response with secretion of chemokines (25). Computational studies indicated that the interaction was guided by hydrophobic contacts between SWCNTs and TLR4, but in the case of carboxylated SWCNTs, the intermolecular interaction was strengthened by short-range electrostatic forces (25). Thus, it appears that the immune system can also “sense” engineered nanomaterials in a manner similar to the sensing of pathogens. However, it is important to distinguish between interactions that are driven mainly by size or shape complementarity (26) vs. those that are defined by specific, molecular interactions. Importantly, as pointed out by Simberg (27), the immune system has evolved to recognize regular arrangements of chemical groups (referred to by immunologists as patterns or motifs) and this is, in essence, what engineered nanomaterials present on their surface. As a case in point, it was shown that the crystalline surface of superparamagnetic iron oxide nanoparticles (SPIONs) can be recognized by the collagen-like domain of the scavenger receptor, SR-A1, and this interaction was sterically hindered by surface polymer coating of the SPIONs (28) (Figure 1). It has been stated that “every human on earth” is exposed to at least one source of carbon-based pollution (29), and it is not unexpected that we have evolved systems to cope with particulates. The fact that immune cells may also sense and engulf engineered nanomaterials through conserved pathways should, therefore, not come as a surprise.

Immune sensing obviously plays a role in vaccination. Luo et al. recently reported that a minimalist, nano-formulated vaccine consisting of a mixture of an antigen and a polymeric nanoparticle was capable of generating cytotoxic T cell responses *in vitro* and *in vivo* (30). The authors provided evidence that this effect was dependent on STING (stimulator of interferon genes), but not on the Toll-like receptor pathway. Evidence was presented for direct binding between the nanovaccine and STING. However, notwithstanding the STING activation evidenced in this study, it remains possible that the NPs also triggered the release of other alarmins thereby boosting anti-tumor immunity. Indeed, STING is a cytosolic DNA sensor, and it is not clear how it recognizes polymeric particles. In another recent study, however, STING-mediated sensing of double stranded DNA (dsDNA) was shown to drive silica-induced lung inflammation (31). Thus, environmental agents such as silica (quartz) may elicit the release of DAMPs, which in turn are sensed by innate immune cells (macrophages or DCs).

## Immune Evasion

Having established that nanomaterials can be recognized and internalized by immune cells, one may ask whether they also can be modified to avoid immune surveillance of the host? This is particularly important in nanomedicine: if the objective is to target, for instance, tumor cells in the brain, then unscheduled clearance by the immune system should be avoided. On the other hand, if the immune system is the target (e.g., antigen delivery for vaccination purposes) then the NPs must be designed with this in mind (7). The most common strategy for avoiding non-specific clearance by the reticulo-endothelial system (RES) and achieving long circulation times involves the grafting of PEG onto the surface of the particles. This reduces, but does not completely abolish, protein adsorption (opsonization or corona formation) on NPs (14). However, while PEGylation is often proposed to improve the stealthiness of NPs, these polymers are not biodegradable, and this may limit their use, in particular if repeated or chronic administration is required. Furthermore, the development of anti-PEG antibodies could lead to accelerated clearance of subsequent doses (15). Schöttler et al. (32) provided evidence that the adsorption of specific proteins could *prevent* cellular uptake of polymer-modified NPs. Hence, the authors used polystyrene NPs that had been modified with PEG or poly(ethyl ethylene phosphate) (PEEP) and demonstrated using mass spectrometry that NPs exposed to human plasma displayed an abundance of clusterin proteins (also known as apolipoprotein J) in their bio-corona. They found that when polymer-modified NPs were incubated with clusterin, non-specific cellular uptake by the murine macrophage-like cell line RAW264.7 could be reduced. Notably, high uptake of NPs in serum-free medium was observed, suggesting that the reduced amount of proteins is not responsible for the inhibition of cellular internalization by “stealth” polymers. However, the latter studies were performed under static *in vitro* conditions. Bertrand et al. (33) recently reported that the enrichment of clusterin/ApoJ on the surface of polymer-based NPs with high PEG densities did not significantly alter their blood circulation time following i.v. administration. It is also possible that proteins may desorb from the surface of NPs upon contact with the blood (34). Clearly, bio-corona formation is a dynamic process and more studies are needed in order to decode the biological “meaning” of the corona.

As already stated, it is important to strike the right balance between cellular uptake by specific target cells and evasion of phagocytic cells of the immune system or RES. Thus, absolute stealth is only of (limited) academic interest. For most nanomedicine applications, cellular uptake with subsequent delivery of the relevant payload is required at some point during the “fantastic voyage” of the NPs through the body. Song et al. (35) provided important insights in a recent study of the cellular “tropism” of poly(lactic acid) (PLA) NPs modified with different polymers. The term “tropism” is borrowed from virology and refers to the manner in which different viruses have evolved to preferentially target specific host species, specific tissues, or specific cell types. Using *in vitro* systems as well as an orthotopic model of glioblastoma, the authors provided an illustrative example of how the surface of NPs can be tuned to modulate cell uptake in healthy and tumor cells, thus highlighting the need to balance cellular uptake and drug release with immune activation and other adverse effects of NPs.

Could NPs be designed such that they avoid unwanted immune clearance in the blood and are available for on-site activation at the desired location, for instance, in a solid tumor? In a recent study, Qiao et al. (36) achieved a reconfigurable nanotherapeutic that is able to shed its PEG shell in the tumor microenvironment in a pH-dependent manner, allowing the dormant cytotoxicity toward the tumor cells to manifest itself. No short-term systemic toxicity was observed in treated animals. This study thus suggests that NPs can be designed simultaneously for immune stealth and on-demand toxicity.

Coating of NPs with PEG serves as a passive means of reducing protein adsorption and avoiding clearance, but as has been discussed in a previous section, this could also hinder uptake of NPs at the desired location; in addition, the polymers may be immunogenic. To circumvent these problems, researchers have employed natural “don't-eat-me” signals such as CD47 in order to furnish NPs with active stealth properties. CD47 is a putative “marker of self” that is expressed on all cell membranes (37). CD47 associates with CD172a, also known as signal regulatory protein-α (SIRPα) on phagocytes and this interaction inhibits macrophage uptake of red blood cells. Interestingly, elevated CD47 expression is co-opted by leukemic stem cells, thereby enhancing their pathogenicity (38). Rodriguez et al. (39) attached minimal “self” peptides computationally designed from human CD47 onto polystyrene NPs, and could show that the self-peptides delayed macrophage-mediated clearance of NPs in mice that were engineered to express a CD172a variant compatible with human CD47. This promoted the circulation time of the NPs and enhanced drug delivery to lung adenocarcinoma xenografts. Furthermore, capitalizing on the discovery of the minimal “self” peptide derived from CD47, Zhang et al. (40) recently developed nano-micelles of poly(lactide-glycolide)-PEG (PLGA–PEG) with stealth properties as a novel theranostic system for simultaneous bioimaging and drug delivery in sarcoma bearing mice.

The complement system is an important part of the innate immune system that *complements* the ability of antibodies to promote the clearance of pathogens and cell debris. Nanomaterials have been shown to activate the complement system through several different pathways, leading to particle opsonisation and clearance (41). However, complement activation may also result in serious adverse reactions, of relevance not least in the context of nanomedicine (42). In addition to complement, nanomaterials may also interact with other soluble proteins belonging to the innate immune system, including the so-called collectins (e.g., surfactant proteins A and D) (43).

In a recent study, Chen et al. showed that superparamagnetic iron oxide “nanoworms” consisting of multiple iron oxide cores surrounded by dextran molecules are opsonized with C3, a protein that fulfills a pivotal role in the activation of the complement cascade (34). The authors showed that the “corona” of adsorbed plasma proteins was located within the dextran shell. Furthermore, they found that C3 covalently bound to these absorbed proteins rather than to the dextran molecules. Surface-bound proteins accelerated the assembly of the complement components of the alternative pathway on the nanoworm surface. C1q, in turn, was originally described as the initiating molecule of the classical complement pathway. However, this protein has wide-ranging roles in immunity not restricted to complement activation (44). Structurally, C1q resembles a bouquet of flowers with six peripheral globular regions each connected by fibrillar strands to a central bundle of fibers. The globular regions are responsible for target recognition while the collagen-like regions mediate immune effector mechanisms, including complement activation and the enhancement of phagocytosis (44). Several studies have shown that C1q binds to nanomaterial surfaces, though this binding does not necessarily lead to complement activation [reviewed in (7)]. Recent modeling studies suggested that C1q is able to disaggregate bundles of multi-walled CNTs, but not those of thin, single-walled CNTs and these predictions were validated with experimental observations (45). These findings may be relevant for the toxicity of CNTs irrespective of whether or not complement is activated, as the (pulmonary) toxicity of CNTs was suggested to be attributable to aggregation of the CNTs rather than the high aspect ratio of individual nanotubes (46). Importantly, the coating of NPs with PEG or other polymers may influence the mode and degree of complement binding (47). The latter study is of particular interest as it suggests that “stealth” approaches that work in a murine environment may not afford immune avoidance in humans.

## Corona Formation

NPs are coated with biomolecules as soon as they are introduced into a biological system (48). Indeed, the adsorption of complement factors such as C1q may be considered as one example. However, very recent studies in which the adsorbed proteins were visualized by using super-resolution fluorescence microscopy have shown that the protein corona is not a dense shell covering the surface of the particle, but instead a heterogeneous network of proteins or clusters of proteins (49, 50). The “corona” concept that was introduced a decade ago (51, 52) has served to focus attention on a crucial aspect of NP interactions with living systems, but one should not *a priori* assume that the NP surface is completely covered by proteins and therefore inaccessible (53). Indeed, it is important to note that in some cases, it is more relevant to consider particle-protein conjugates, and not a “corona” of proteins covering the surface of the NP. For instance, Deng et al. (54) examined the binding of fibrinogen, a large cylindrical molecule of 45 nm in length, to negatively charged poly(acrylic acid)-coated gold NPs ranging in size from 7 to 22 nm. Each fibrinogen molecule could accommodate two 7 nm particles, but only one when the diameter of the NP was increased to 10 nm. The authors found that particles larger than 12 nm bound multiple fibrinogen molecules. However, in the presence of an excess of NPs, fibrinogen induced aggregation of the latter particles suggestive of interparticle bridging (54). This could have ramifications for immune responses to NPs, as the immune system is geared toward the recognition of conjugates of small molecules (haptens) with proteins.

To date, the vast majority of bio-corona studies have been conducted *in vitro* (55–57) and while this has served to underscore the importance of the acquired biological “identity” of nanomaterials as they encounter a biological environment, the *in vivo* relevance has remained obscure. However, in the past few years, several studies on *in vivo* bio-corona formation have emerged, thus shedding light on the impact of the bio-corona on NP clearance or targeting (58, 59), and its role for the toxicological outcomes of NP exposure (34, 60). However, it is important to realize that differences in kinetics of NP clearance in humans vs. smaller species such as mice may result in more or less pronounced effects of bio-corona formation, and this needs to be taken into consideration when making interspecies extrapolations about the biodistribution of NPs (61). Moreover, the overwhelming majority of all bio-corona studies to date have focused on the protein corona, while other biomolecules including lipids or nucleic acids have been somewhat neglected. However, lipids, in particular, deserve special attention as they are key constituents of the cell membrane, and are involved in numerous signaling pathways. Hellstrand et al. (50) reported almost a decade ago that copolymer NPs [50:50 *N*-isopropylacrylamide (NIPAM):*N*-*t*-butylacrylamide (BAM)] bind cholesterol, triglycerides and phospholipids from human plasma, and noted that the lipid and protein binding patterns corresponded closely to the composition of natural, high-density lipoprotein (HDL) complexes. HDL particles are nano-sized protein complexes that transport lipids in the body, the most abundant apolipoproteins in HDL particles being Apo-AI and Apo-AII. It is pertinent to note that apolipoproteins are frequently detected in the bio-corona of various NPs (62, 63). The results of the aforementioned study implied that such NPs may be recognized by living systems as HDL complexes, and that nanoparticles may exploit existing transport pathways for lipoprotein particles (64), underscoring that recognition and handling of NPs may transpire *via* conserved pathways. In a recent study, Lara et al. (65) provided evidence, using an elegant immuno-epitope mapping approach, that two major proteins in the serum corona, low-density lipoprotein and immunoglobulin G, present functional motifs to allow simultaneous recognition by low-density lipoprotein receptor (LDLR) and Fc-gamma receptor I, respectively. Collectively, these findings suggest that NPs may be “mistaken” for endogenous particles, such as lipoproteins, and exogenous entities, such as viruses, by virtue of specific components of the bio-corona (65). Does this have any implications for the *in vivo* fate of NPs? In a recent study using NPs prepared from poly(ethylene glycol)-*b*-poly(lactic co glycolic acid) (PEG–PLGA) copolymers, Bertrand et al. (33) could show that the adsorption of apolipoprotein E (ApoE) following intravenous injection of the NPs into mice appeared to be dependent on PEG density; the authors also found that for NPs with low PEG coverage, adsorption of apolipoproteins could prolong circulation times. In addition, the LDLR was found to play a key role in the clearance of NPs, irrespective of PEG density. Thus, it appears that ApoE exerted distinct functions on NPs with low and high PEG densities. Notwithstanding, these findings suggest that apolipoproteins involved in the trafficking of lipids in the bloodstream also impact on the *in vivo* clearance of NPs.

Once inside the cell, it is presumed that the protein corona is removed in phagolysosomes, causing the true “identity” of the NPs to be revealed (66). Wang et al. (67) showed, using positively charged polystyrene NPs, that the adsorbed protein corona is retained on the NPs as they enter cells and are trafficked to the lysosomes. There, the corona is degraded and this is followed by lysosomal damage, leading to the release of lysosomal proteases (cathepsins) into the cytosol, and apoptosis. In a subsequent study, it was shown that the intracellular degradation of proteins that are ferried into cells by NPs is different compared to what is observed when proteins are transported freely into cells (68). One may ask whether NPs can also acquire a new bio-corona inside the cell, for instance following their escape from the lysosomal compartment, or following translocation across the plasma membrane by non-endocytotic pathways with direct access to the cytoplasm (69), or whether intracellular stealth is possible? Sund et al. (70) reported that metal oxide NPs bound several ribosomal and cytoskeletal proteins upon incubation with cytoplasmic extracts of macrophages, and binding was more effective for nano-sized TiO_2_ NPs when compared to the coarse form (5 μm) of TiO_2_. In a recent study, semisynthetic, magnetic nanoparticles based on the natural protein cage ferritin were produced, and the authors observed “cytosolic stealth” (i.e., avoidance of intracellular degradative processes) as a function of PEGylation of the particle surface; non-PEGylated particles co-localized with autophagosomes upon microinjection into cells (71). Understanding and controlling these processes would be of considerable importance for biomedical applications of NPs: what good are NPs if they are stuck in lysosomes or autophagosomes and cannot deliver their cargo? Can we learn from immunology and microbiology in this regard? Viruses—natural nano-scale particles—use a variety of different strategies to escape from the host immune system (72) and so do bacteria (73). However, it gets even more complicated: a recent study showed that nano-sized particles, but not microparticles, associated with fungal spores, and “coronation” of the human pathogen, *Aspergillus fumigatus* with synthetic NPs affected its pathobiological behavior (74). One may speculate that such NP-pathogen hybrids could display distinct molecular patterns that trigger novel or unanticipated biological responses (75). On the other hand, the spores of *Aspergillus fumigatus* are surrounded by a corona of hydrophobin that masks the underlying cell-wall polysaccharides, making them immunologically inert (76). Natural stealth solutions from the microbial kingdom hold exciting prospects, and hydrophobin is being applied in the development of NPs or supraparticles (77–79).

## Targeting—the Holy Grail

Targeted or “smart” delivery of drugs is arguably the Holy Grail of pharmacology (80). Nanomedicine offers one possible way to achieve this elusive yet important goal, and numerous studies have been published on NPs functionalized with ligands that should guide the particles and their payload preferentially to diseased tissues (81, 82). However, relatively few actively targeted nanomedicines have reached clinical trials. It has been suggested a few years ago that drug delivery researchers ought to look more closely at how viruses and bacterial toxins exploit the cellular machinery of the host to gain access to intracellular targets (83). The author(s) also pointed out that these microbial agents have spent “a few million years more” than drug developers learning how to enter cells. On the other hand, others have suggested that we need to develop *less* sophisticated delivery technologies that build on robust physicochemical or biological principles more amenable to clinical translation (84). Indeed, adding functionality to NPs could potentially lead to a more convoluted or unpredictable behavior *in vivo*, as well as greater regulatory hurdles (85). Nevertheless, there seems to be a delivery problem (86). Wilhelm et al. recently concluded, on the basis of a meta-analysis of the literature on targeted NPs for cancer treatment, that only 0.7% (median) of the administered NP dose is actually delivered to the tumor (87). However, this has stirred up a storm: not everyone agrees that nanomedicines should be judged by the number of particles that are present in the tumor (88), while other investigators have pointed out that even one percent “can actually be useful if it happens in the right cells” (89). In a recent follow-up study, <14 out of 1 million (0.0014% injected dose) intravenously administrated NPs were delivered to cancer cells, and only 2 out of 100 cancer cells interacted with the NPs. The majority of the intratumoral NPs were trapped in the extracellular matrix or taken up by perivascular tumor-associated macrophages or TAMs (90).

**Figure 2:**
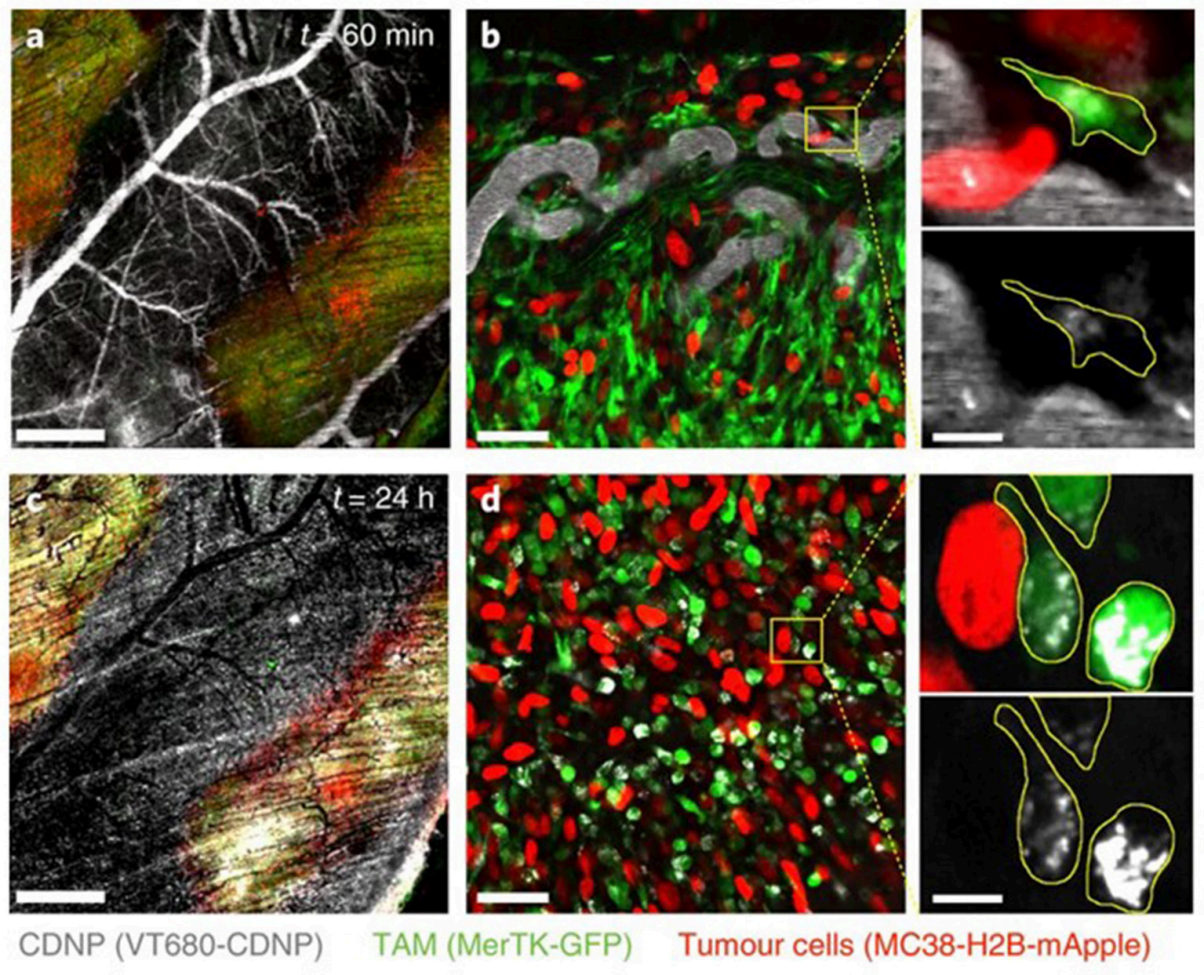
Uptake of NPs by tumor-associated macrophages. Rodell et al. (93) examined the distribution of fluorescent cyclodextrin nanoparticles (CDNPs) by confocal fluorescence microscopy in MerTK-GFP mice bearing a colorectal tumor (MC38) **(a)**. In these reporter mice, tumor-associated macrophages (TAMs) are readily detectable based on GFP expression. High-magnification images **(b)** demonstrated rapid CDNP accumulation in perivascular macrophages. Vascular clearance was observed 24 h post-injection **(c)**, and CDNPs were well-distributed to TAMs **(d)**. Refer to Rodell et al. (93) for details. Reprinted from Rodell et al. (93) with permission from Springer Nature.

The question, then, is whether one should re-think the immune “stealth” approach for delivery of nanomedicines and aim for specific immune cell populations as a means of hitchhiking into tumors: “if you can't beat them, join them.” Smith et al. reported that SWCNTs are almost exclusively taken up by a specific immune cell subset, namely Ly-6C^high^ monocytes expressing the surface glycoprotein lymphocyte antigen 6C (Ly-6C), and subsequently delivered to tumors in mice (91). The uptake mechanism, and whether any specific cell surface receptors were involved, was not disclosed. However, the remarkable selectivity suggests that NPs can be delivered to tumors *via* certain subsets of circulating blood cells through a Trojan horse mechanism. Furthermore, Choi et al. reported a decade ago that gold nanoshells are phagocytosed by both monocytes and macrophages, and showed infiltration of these cells in a human breast tumor spheroid and photo-induced cell death in the hypoxic microenvironment of the spheroid (92). Overall, targeting of TAMs represents one possible approach to the problem of drug delivery to tumors, though unscheduled clearance by “competing” macrophages in other organs such as the liver, lungs, and spleen needs to be avoided (94). In a recent study, Rodell et al. leveraged macrophage affinity for cyclodextrin-based NPs to achieve efficient TAM delivery (Figure 2), preferentially altering the phenotype of the macrophages; when used in combination with the immune checkpoint inhibitor, anti-PD-1 the authors observed an improved tumor response to immunotherapy (93). Hence, the propensity of macrophages to engulf particles can be harnessed to deliver immune-modulating drugs that promote anti-cancer activity (95).

Much of the literature on phagocytic uptake and clearance of NPs is focused on macrophages, but it is important to realize that other phagocytic cells, including neutrophils, are also capable of internalizing NPs (96). Again, one may ask whether neutrophil clearance could be exploited for therapeutic gain? Wang et al. reported that drug-loaded albumin NPs are internalized by neutrophils adherent to the activated endothelium *via* cell surface Fcγ receptors (97), suggesting new avenues to treat a broad range of inflammatory diseases. Similarly, Chu et al. reported a strategy for delivering therapeutic NPs across the blood vessel barrier by allowing NPs to “hitchhike” with activated neutrophils (98). The authors demonstrated that intravenously infused albumin NPs were specifically internalized by activated neutrophils, and the NP-containing neutrophils subsequently migrated across blood vessels into the inflamed tissues. Drug-loaded albumin NPs markedly ameliorated the lung inflammation induced by lipopolysaccharide or infection by *Pseudomonas aeruginosa* (98).

## Future Perspectives

Deciphering the immunological interactions of nanomaterials with or without a corona of proteins, lipids, and other biomolecules remains a formidable task. However, perhaps we have been barking up the wrong tree. Instead of asking whether NPs exert new and unanticipated effects when compared to the same materials in their bulk form, perhaps one should ask whether there are any lessons from previous studies of other (natural) nano-sized objects including viruses and other pathogens. Hence, while the discipline of nanotoxicology builds on studies of other fine and ultrafine particles and fibers (99), important lessons may also be learned from immunology in terms of how the immune system senses and handles foreign objects (100). Conversely, one may learn from the sophisticated immune evasion or immune targeting strategies evolved by pathogens and apply these lessons in the design of biocompatible nanomedicines.

DNA is perhaps the ultimate nanomaterial (101) and so-called DNA origami, i.e., the purposeful folding of DNA to create non-arbitrary two- and three-dimensional shapes, is rapidly gaining traction, and may find use in drug delivery and other medical applications. Yet one may ask how readily does a naked DNA-based delivery system negotiate the extracellular environment, and how efficient is the cellular uptake; besides, what could be more immunogenic than foreign, naked DNA? Perhaps the solution lies in encapsulating these DNA structures in protein envelopes (102, 103). Thus, it appears that we have come full circle, with the development of artificial, nano-scale systems that mimic natural nano-scale particles (i.e., viruses) (104). Other natural nanoparticles are also attracting attention for their potential biomedical applications. For instance, the cytoplasmic ribonucleoprotein known as the vault, named for its appearance with multiple arches reminiscent of cathedral ceilings, is currently studied as a platform for a wide range of therapeutic applications including drug or antigen delivery (105, 106). Other biomimetic particles cloaked in cell membranes (so-called leukosomes) are also being developed for biomedical purposes (107, 108), along with naturally occurring extracellular vesicles (exosomes) (109). Furthermore, in an intriguing study, biohybrid “microrobotic” entities based on naturally fluorescent microalgae were produced *via* a dip-coating process in magnetite (Fe_3_O_4_) suspensions for the purpose of imaging-guided therapy (110). Overall, the development of semisynthetic “cyborg” particles based on templates drawn from viruses or other pathogens, or inspired by endogenous intra- or extracellular particles, all of which deploy strategies of immune recognition and/or immune evasion that have been honed by evolution, could potentially allow for novel, biocompatible systems for imaging and drug delivery.

## Author Contributions

The author confirms being the sole contributor of this work and has approved it for publication.

### Conflict of Interest Statement

The author declares that the research was conducted in the absence of any commercial or financial relationships that could be construed as a potential conflict of interest.
